# Improving Identification of *In-organello* Protein-Protein Interactions Using an Affinity-enrichable, Isotopically Coded, and Mass Spectrometry-cleavable Chemical Crosslinker[Fn FN1]

**DOI:** 10.1074/mcp.RA119.001839

**Published:** 2020-02-12

**Authors:** Karl A. T. Makepeace, Yassene Mohammed, Elena L. Rudashevskaya, Evgeniy V. Petrotchenko, F.-Nora Vögtle, Chris Meisinger, Albert Sickmann, Christoph H. Borchers

**Affiliations:** ‡ Department of Biochemistry and Microbiology, University of Victoria, 3800 Finnerty Rd., Victoria, BC V8P 5C2, Canada; § University of Victoria - Genome British Columbia Proteomics Centre, #3101-4464 Markham Street, Vancouver Island Technology Park, Victoria, BC V8Z7X8, Canada; ¶ Center for Proteomics and Metabolomics, Leiden University Medical Center, Albinusdreef 2, 2333 ZA, Leiden, The Netherlands; ‖ Leibniz Institut für Analytische Wissenschaften - ISAS - e.V., Dortmund, Germany; ** Segal Cancer Proteomics Centre, Lady Davis Institute, Jewish General Hospital, McGill University, Montreal, Quebec, H3T 1E2, Canada; ‡‡ Institute of Biochemistry and Molecular Biology, ZBMZ, Faculty of Medicine, University of Freiburg, Freiburg, Germany; §§ Signalling Research Centres BIOSS and CIBSS, University of Freiburg, Germany; ¶¶ Gerald Bronfman Department of Oncology, Jewish General Hospital, Montreal, Quebec, H3T 1E2, Canada; ‖‖ Department of Data Intensive Science and Engineering, Skolkovo Institute of Science and Technology, Skolkovo Innovation Center, Nobel St., Moscow 143026, Russia

**Keywords:** Crosslinking, protein-protein interactions, mass spectrometry, mitochondria function or biology, protein complex analysis, organelle-wide interactions

## Abstract

An experimental and computational approach for identification of protein-protein interactions by *ex vivo* chemical crosslinking and mass spectrometry (CLMS) has been developed that takes advantage of the specific characteristics of cyanurbiotindipropionylsuccinimide (CBDPS), an affinity-tagged isotopically coded mass spectrometry (MS)-cleavable crosslinking reagent. Utilizing this reagent in combination with a crosslinker-specific data-dependent acquisition strategy based on MS2 scans, and a software pipeline designed for integrating crosslinker-specific mass spectral information led to demonstrated improvements in the application of the CLMS technique, in terms of the detection, acquisition, and identification of crosslinker-modified peptides. This approach was evaluated on intact yeast mitochondria, and the results showed that hundreds of unique protein-protein interactions could be identified on an organelle proteome-wide scale. Both known and previously unknown protein-protein interactions were identified. These interactions were assessed based on their known sub-compartmental localizations. Additionally, the identified crosslinking distance constraints are in good agreement with existing structural models of protein complexes involved in the mitochondrial electron transport chain.

## Abstract (precis)

By using an enrichable, isotopically labeled, MS-cleavable crosslinking reagent, a targeted MS2 acquisition strategy, and a novel software pipeline tailored to integrating crosslinker-specific mass spectral information we improved the detection, acquisition, and identification of crosslinker-modified peptides. Our method applied to isolated yeast mitochondria allowed us to observe protein-protein interactions involving approximately one quarter of the proteins in the mitochondrial proteome. Our approach is suitable for proteome-wide applications, and facilitates investigations into condition-specific protein conformations, protein-protein interactions, system-wide protein function or dysfunction, and diseases.

## Graphical Abstract

**Figure:**
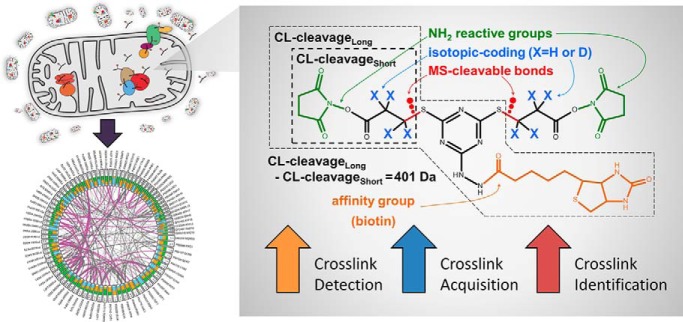

- Used affinity-enrichable, isotopically coded, and MS-cleavable crosslinker.
- Targeted acquisition strategy based on isotopic-coding described and evaluated.
- Novel data analysis pipeline developed provides improved crosslink identification.
- Large dataset reveals hundreds of mitochondrial protein-protein interactions.

Proteins and their intricate networks of interactions are fundamental to many of the molecular processes that govern life (1, 2). Insights into the structures of individual proteins and their interactions with other proteins in a proteome-wide context has been made possible by recent developments in the relatively new field of chemical crosslinking combined with mass spectrometry (CLMS)^1^ (3, 4). Crosslinkers stabilize transient interactions by forming covalent chemical linkages between amino acid residues. The crosslinked proteins are then enzymatically digested into peptides, and the covalently coupled crosslinked peptides are identified by mass spectrometry. These identified crosslinked peptides thus provide evidence of interacting regions within or between proteins (5–13).

Proteome-wide crosslinking analysis has the potential to provide structural characterization of protein-protein interactions and protein complexes in their natural cellular and tissue environments. Moreover, the technique is well suited for capturing the “molecular sociology” of the cell, including the more weakly interacting and transient complexes. Such interactions may not be identified through traditional biochemical techniques using rigorous purification procedures that tend to only be compatible with robust complexes (1, 14).

Although this technique is straightforward, for proteome-wide applications it is made considerably more complex by the combinatorial nature of the crosslinked peptides, which can originate from any of the proteins in the proteome. To address this issue, cleavage of the crosslinker itself—which then provides information on the masses of the individual peptides constituting a crosslink—has been recognized as a critical feature for the crosslinking analyses of complex samples (13, 15–21). Several successful analytical strategies exploiting this feature have recently been reported for proteome-wide crosslinking studies (13, 22, 23). The relative and absolute abundances of crosslinked peptides in typical peptide digests are much lower than those of single peptides, so specific enrichment of crosslinked peptides from the total peptide digest has also been shown to be critical for successful analyses (20). Another advantageous feature that may be incorporated into the crosslinker is isotopic coding. It enables specific selection of the crosslink signals in MS1 for subsequent MS/MS analysis, and adds additional characteristic features to the spectra of the crosslinks, which can then be used to further improve the confidence of the identification (20).

Here we report the application of the affinity-enrichable isotopically coded and CID-cleavable crosslinker cyanurbiotindipropionylsuccinimide (CBDPS) to *in-organello* crosslinking analysis (20). We describe a CLMS workflow that improves upon previously published workflows in terms of detection, acquisition, and identification of crosslinked peptides (22, 24–26). This study yielded a rich crosslinking dataset, revealing hundreds of intra- and inter-molecular protein-protein interactions within the mitochondrial organelle. Using this analytical approach, we have uncovered system-wide interaction patterns that would not be accessible through classic protein-chemistry research techniques.

## EXPERIMENTAL PROCEDURES

### 

#### 

##### Materials and Reagents

All materials were from Sigma-Aldrich, St. Louis, MO, unless noted otherwise.

##### Mitochondria Preparation and In-organello Crosslinking

Highly purified yeast mitochondria, strain YPH499, were prepared as described previously (27, 28). The mitochondrial sample was thawed on ice, and then diluted gently to 5 mg/ml in isotonic buffer (250 mm sucrose, 1 mm EDTA, 10 mm MOPS-KOH, pH 7.2). Mitochondria were crosslinked with an equimolar mixture of isotopically light and heavy cyanurbiotindipropionylsuccinimide (CBDPS-H8 and CBDPS-D8, respectively) (Creative Molecules, Inc., Montreal, Quebec, Canada) at 2 mm as follows: samples were pre-warmed at 21 °C for 5 min; after addition of the crosslinker mixture, samples were kept at 21 °C for 10 min and then put on ice for 110 min. The crosslinking reaction was quenched with the addition of ammonium bicarbonate to a final concentration of 50 mm for 20 min. Crosslinked mitochondria were collected by centrifugation at 18,000 × *g* for 20 min in the cold, and immediately lysed.

##### Sample Lysis, Prefractionation and Digestion

The pellet of crosslinked mitochondria was resuspended in a hypotonic buffer consisting of 1 mm EDTA, 10 mm MOPS-KOH, pH 7.2, left on ice for 20 min and lysed by sonication using a Vibra Cell Ultrasonic Processor for a total processing time of 1 min (70% amplitude, 5 pulses). The lysate was centrifuged at 18,000 × *g* for 20 min, and the resulting pellet (Pellet1) and supernatant were collected, frozen in liquid nitrogen and stored at −80 °C until the next day. Pellet1 was used to prepare all of the samples, and is hereafter referred to as “membrane1” or “membrane low centrifugation.” The supernatant was centrifuged at 100,000g for 45 min and the resulting pellet (Pellet2) and supernatant used to prepare all the samples are hereafter referred to as “membrane 2” or “membrane high centrifugation” and “soluble,” respectively. Proteins were solubilized from Pellet 1 and Pellet 2 with 2% SDS in 10 mm MOPS-KOH pH 7.2, at 37 °C for 30 min and 300 rpm, with subsequent centrifugation at 18000 × *g* for 20 min.

Proteolysis was performed with trypsin (Promega, Madison, WI, Sequencing Grade Modified, trypsin/protein ratio 1:20) using the FASP protocol (29) with modifications and ultrafiltration units with a nominal molecular weight cutoff of 30 kDa (Vivacon^®^ 500, Sartorius, Goettingen, Germany). Samples were loaded to prewashed filtration units (≤ 400 μg of protein per unit). After preconcentration, samples were washed with 400 μl of 8 m urea buffer, treated with 200 μl 0.1 m DTT solution, 200 μl 0.05 m IAA solution, washed 3× with 200 μl 8 m urea solution, 3× with 50 mm Tris-HCl buffer pH 8.5. Digestion was performed overnight (18 h) at 37 °C. Peptides were collected by washing the filter units with 100 μl 50 mm Tris-HCl buffer pH 8.5 and then 200 μl 0.5 m NaCl.

##### Enrichment of Crosslinked Peptides

The resulting peptide mixture was acidified with formic acid, desalted using C18 SPE columns (BondElute SPEC C18AR, Agilent Technologies, Santa Clara, CA), eluted with 0.4% formic acid with 90% acetonitrile, and dried completely. Samples were reconstituted with SCX buffer A (10 mm KH_2_PO_4_, 20% acetonitrile, pH 2.7), and separated by strong cation exchange (SCX) chromatography using an UltiMate 3000 HPLC system and an POLYSULPHOETHYL A column (PolyLC INC, Columbia, MD, 5 μm particle size, 200Å pore size, 150 × 1.0 mm) (30). A ternary buffer system was used: SCX buffer A (10 mm KH_2_PO_4_, 20% acetonitrile, pH 2.7), SCX buffer B (10 mm KH_2_PO_4_, 250 mm KCl, 20% acetonitrile, pH 2.7) and SCX buffer C (10 mm KH_2_PO_4_, 600 mm KCl, 20% acetonitrile, pH 2.7). From each sample, 19 SCX fractions were collected at 37.5–250 mm KCl and dried. Collected fractions were further enriched for CBDPS crosslinked peptides on monomeric avidin beads (Pierce Biotechnology) as described previously (24) and analyzed by LC-MS/MS.

##### LC-MS/MS Analysis

Mass spectrometric analysis was performed using a Dionex UltiMate3000 (Thermo Fisher Scientific, Waltham, MA) coupled to the ESI-source of an Orbitrap Fusion Lumos or Q Exactive HF (Thermo Fisher Scientific). Samples were loaded in 0.1% TFA onto a trapping column (Acclaim PepMap 100 C18, 5 μm particle size, 100 μm × 2 cm, Thermo Scientific) for pre-concentration. Peptides were separated on C18 analytical column (Acclaim PepMap RSLC, 75 μm × 500 mm, 2 μm, 100 Å, Thermo Fisher Scientific) using a binary gradient (solvent A: 0.1% formic acid (FA); solvent B: 0.1% FA, 84% ACN). For MS analysis on the Lumos, peptides were separated with a 120-min gradient (0–100 min: 3–35% solvent B (84% acetonitrile, 0.1% FA), 100–110 min: 35–42% B, 110–120 min : 42–80% B, 0.250 μl/min flowrate). On the Q Exactive HF, peptides were separated with 180 min gradient: 0–160 min: 3–35% solvent B, 160–170 min: 35–42% B, 170–180 min 42–80% B.

MS data were acquired using data-dependent methods utilizing either TopSpeed (TopS) or TopN; targeted mass difference (MTag); or inclusion list (Incl) precursor selection modes (24).

##### Data-dependent Acquisition Methods

The data-dependent acquisition utilized dynamic exclusion, with an exclusion duration of 30 s and exclude after n times set to 1 (Lumos). MS and MS/MS events used 120,000 and 60,000 resolution FTMS scans, respectively, with a scan range of 350–1800 *m*/*z* in the MS mode. For the TopN methods a loop count of 10 was used. For the TopS method, a cycle time of 3 s was used. For MS/MS acquisition, the HCD collision energy was set to 28% NCE for Q Exactive HF runs and CID of 35% for Orbitrap Fusion Lumos runs. Only precursor ions with charge states of +3 to +7 were selected for fragmentation. The acquisition method for targeted mass difference (MTag) runs was identical to the method described for the TopS acquisitions except that a “Targeted Mass Difference” filter with the mass difference set to 8.0502 Da with a light-heavy analogue intensity range set to 50–100 was used. The acquisition method for inclusion list (Incl) runs was identical to the method described for the TopS acquisitions except that a “Targeted Mass” filter was used. The parent mass lists used in the “Targeted Mass” filter for these analyses were calculated using Hardklör (ver. 2.3.0; see supplemental Table S1 for parameters (31), Krönik (ver. 2.02; see supplemental Table S2 for parameters) (32), and in-house scripts. Only doublets that were identified as charge state 3 and greater were included in the parent mass list.

##### MS1 Feature Analysis

The identification of doublets (Δ8.0502 Da) in MS1 and evaluation of crosslinker-modified precursors was accomplished using Hardklör, Krönik, and in-house scripts. Criteria for classifying an MS1 feature from the Kronik output as a doublet was that the light and heavy monoisotopic peaks for MS1 features were separated by 8.0502 Da ±0.01 Da, that the heavy-isotopic peak had a maximum intensity that occurred at a retention time that is between −0.4 min and 0.05 min of the maximum intensity of the light-isotopic peak, that the log_2_ of the heavy-isotopic partner summed intensity divided by the light-isotopic partner summed intensity was 0 ± 2, and that the maximum intensities observed for both the light and the heavy isotopic peaks are each greater than or equal to 25000 intensity units.

##### Bioinformatics Analysis

RAW data files were converted to mzXML format using MSConvertGUI (v.3.0.10730) of the ProteoWizard tool suite (release 3.0.11252) (33) and the data analysis was completed using our Qualis-CL software pipeline (manuscript in preparation). The pipeline consists of 5 external open source software packages and 4 in-house developed modules to allow crosslinked peptides identification, MS1 and MS2 feature annotation, and validation.

Inter, intra and loop Lys-Lys crosslinked peptides as well as single peptide and protein group identifications were obtained using Kojak search engine (ver. 1.5.5) (see supplemental Table S3 for parameters) (34). In its diagnostic mode, Kojak reports detailed results on how the (crosslinked) peptides were assigned to each spectrum. This was an essential aspect as we made use of this detailed information in our pipeline. The database for data analysis included list of proteins identified earlier in highly purified mitochondrial samples (35), and proteins that have reference to mitochondria in their description in Saccharomyces Genome Database (SGD) and/or UniProt. Thus, it contained known mitochondrial proteins, associated proteins and contaminants. The database included concatenated target and decoy protein sequences, in which the decoy entries were generated by shuffling each peptide's amino acids in each protein target entry using our own algorithm (supplemental material 1). This generates decoy entries that have distributions of protein and peptide lengths that are similar to the target proteins. The database contains 1295 protein entries, and same number of decoy entries. All searches were performed with carboxyamidomethylated cysteine as a fixed modification, methionine oxidation as a variable modification and a maximum of 3 tryptic missed cleavages.

Hardklör (31) and Krönik (32) software tools were used to determine MS1 spectral and chromatographic features associated with the MS1 parent masses that had been acquired with MS2 in the raw data. The MS1 features detected by Hardklör and Krönik software tools were then used as input for our in-house algorithm to find those features that exist as multiplets (doublets, triplets, or quadruplets) within user-specified tolerance settings between the heavy and light pairs. The tolerances allow for variations in retention times between the labeled and unlabeled pairs, relative intensity differences (20% in this work), and variations in the mass differences of the doublets (0.01 Da here).

The MS2 features that result from cleavage products of the crosslinker were detected and annotated using an algorithm written in-house. For each assigned MS2 spectrum, we determined the presence of 4 crosslinker cleavage products. Following calculation of MS1 and MS2 features additional logic calculates meta-features that check for agreement between MS1 and MS2 features.

Identifications, scores, MS1 features, MS2 features, and meta-features (described in supplemental Table S4) were combined in one table per crosslink type, *i.e.* inter-protein, intra-protein, loop, or single. The Percolator algorithm (ver. 2.08) was used to perform the validation and the calculation of the q-values (see supplemental Table S5 for parameters). All software used was combined into a single pipeline that takes raw data in mzXML format and generates the result tables. An additional module combines these result tables with interactome databases to generate statistics and highlights the known and novel interaction.

The software modules developed in-house are available from the authors as well as online at http://bioinformatics.proteincentre.com/Qualis-CL/.

##### Structural Validation of Crosslink Identifications

XiView (36) and open source PyMOL (37) were used to map crosslinks to existing structural models for yeast electron transport chain complexes and super-complexes.

##### Experimental Design and Statistical Rationale

Crosslinked sample fractionation into one soluble and two membrane fractions was performed to allow commenting on the sub-compartment localizations of the detected protein interactions. Each sample fraction sample was digested and further separated by SCX chromatography. Each SCX chromatographic sample for the soluble fractions was analyzed with 3 different acquisition strategies allowed by the instrument software, these were: (1) data dependent acquisition of the top 10 features or for 3 s - TopS/TopN, (2) triggering on the presence of mass difference of two MS1 features equal to the difference of heavy and light crosslinker pairs - Mtag, and (3) inclusion list based on post analysis of the TopS/TopN and Mtag data - Incl. Both membrane fractions were analyzed with TopS only. These multiple analyses of fractions are complementary replicates. Biological replicates would be prohibitively expensive in terms of the total number of LCMS samples used for the experimental design of this study. The decoy entries in the search database were generated by randomizing the sequence while keeping the C terminus amino acid unchanged for all tryptic peptides in each protein. This ensures decoy entries which are very similar to the forward ones in terms of the number, length, and composition of the tryptic peptides. q-values were estimated by Percolator software and were used for all FDR thresholds. FDR cutoff value was put at 2% at the identified crosslinked peptide level.

## RESULTS

### 

#### 

##### Developing an Integrated Experimental and Computational Crosslinking-MS Workflow

**Fig. 1.**
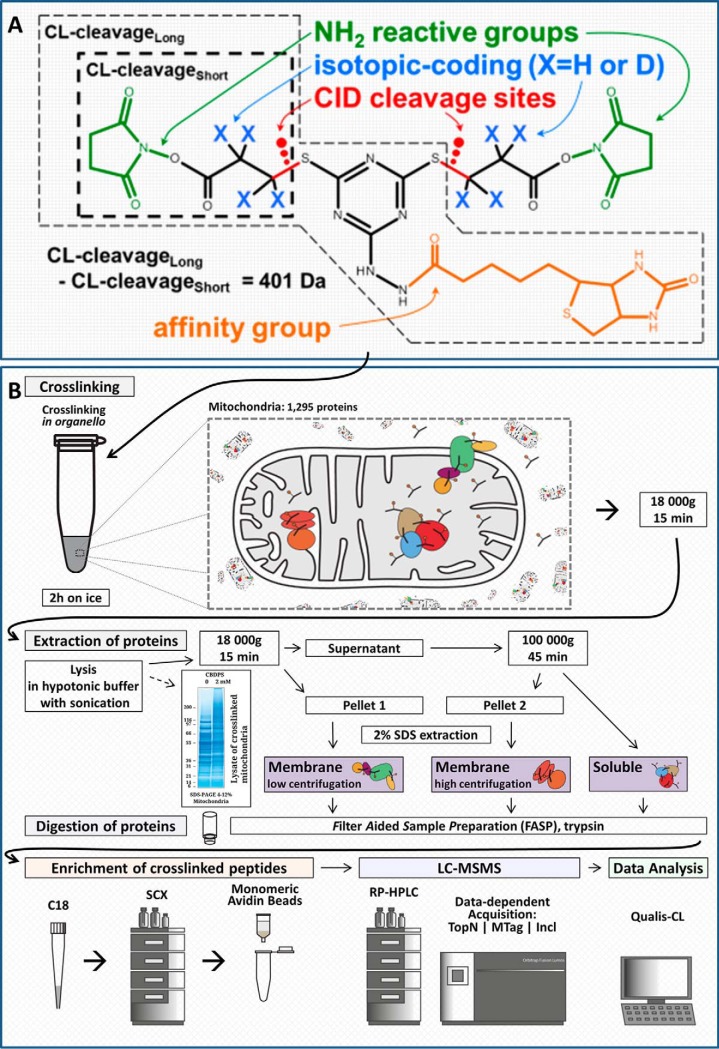
**Crosslinking reagent and experimental workflow.** *A*, CBDPS molecular diagram showing NH_2_ reactive groups, CID-cleavable bonds, isotopic-coding positions, and biotin affinity-tag. Short and long crosslinker-cleavage portions are also indicated. *B*, General experimental workflow for *in-organello* crosslinking. The affinity enrichment, LCMS analysis, and data analysis steps all take advantage of the various features of the CBPDS crosslinker shown in (*A*). Specifically, the biotin-tag of the crosslinker allows the enrichment of crosslinker-modified peptides prior to MS analysis, the isotopic-labeling allows the use of targeted MS acquisition methods, and the mass spectral; features relating to both the isotopic-labeling and crosslinker-cleavage result in improved confidence in peptide-spectrum match identifications.

Previously, we developed a multifunctional crosslinking reagent, CBDPS, that combined several features which improve the performance of CLMS analyses: affinity-enrichability, isotopic-coding, and MS-cleavability (20). By taking advantage of the specific biochemical and physical features of the CBDPS crosslinking reagent (Fig. 1*A*), we were able to improve the detection, acquisition, and identification of crosslinker-modified peptides in a complex sample. These improvements affect three critical points in the analytical workflow (Fig. 1*B*): (1) affinity-enrichment of crosslinker-modified peptides; (2) specific MS2 acquisition of crosslinker-modified peptides using targeted mass difference (MTag) or inclusion list (Incl) data-dependent acquisition methods; (3) use of crosslinker-specific spectral features in the validation of crosslinks. Here we utilize this reagent for proteome-wide analyses by taking advantage of these features in both the experimental and computational aspects of the CLMS workflow.

##### Affinity Enrichment for Improved Detection of Crosslinker-modified Peptides

**Fig. 2.**
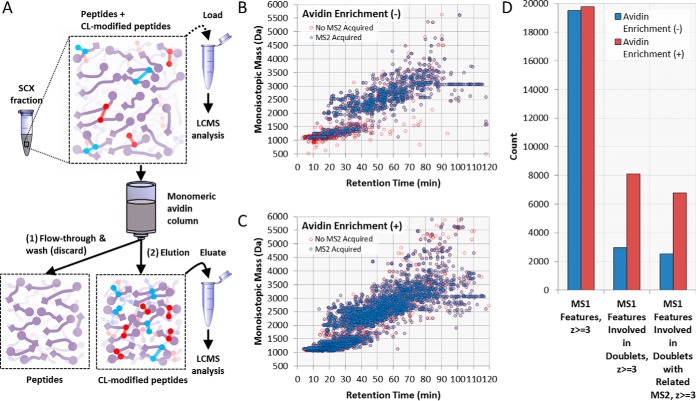
**Affinity enrichment improves detection of crosslinker-modified peptides.** *A*, Diagram of the affinity-tag-based enrichment strategy. An SCX fraction containing a mixture of peptides and crosslinker-modified peptides is loaded onto a monomeric avidin column. Peptides that do not contain crosslinker are discarded in the flow-through and wash fractions whereas those that do are retained. These retained crosslinker-modified peptides are eluted from the column and collected (eluate) for subsequent LCMS analysis. A portion of the SCX fraction prior to enrichment (load) may also be saved for LCMS analysis to assess the improvement in crosslinker-modified species detected as shown in *B*. A comparison of Δ8.0502 Da doublet features found in MS1 for samples without (load) (*B*) and with (eluate) (*C*) enrichment shows ∼2.7 times as many doublet features with enrichment (*D*).

The yield of crosslinking products is typically low; therefore enrichment procedures prior to mass spectrometry analysis dramatically improve the detection and identification of these crosslinks (Fig. 2*A*). Specific enrichment of CBDPS crosslinker-modified peptides is achieved using the biotin tag which has been incorporated into the reagent enabling enrichment with avidin. It should be noted that inter-peptide crosslinks and single peptides containing CBDPS “dead-end” or “loop-link” modifications would both be enriched. For this reason, a chromatographic step to separate inter-peptide crosslinks from single peptides (*e.g.* strong cation exchange chromatography (38) or size exclusion chromatography (39)) is often performed prior to affinity enrichment, and can further assist in the CLMS analysis. In order to quantitate the resulting improvement in detection of crosslinker-modified peptides, we compared the number of MS1 doublet features (supplemental Fig. S1) that were observed in crosslinked samples before and after enrichment. Almost twice as many (170%) CL-modified MS1 features were detected after the affinity-tag enrichment procedure (Fig. 2*B*).

##### Isotopic-coding for the Specific Acquisition of Crosslinker-modified Peptides

In our initial analyses, a distinct bimodal distribution in the TopN spacing (*i.e.* the number of MS2 scans occurring between MS1 scans) was observed for the SCX fractions that were expected to be most abundant in crosslinker-modified peptides (supplemental Fig. S2). This indicated to us that the duty cycle was frequently reaching its maximum allowed time duration between consecutive MS1 scans and may, therefore, be unable to acquire all the unique precursors at those particular retention times. The time-dependent effects of the TopN spacing indicated that the duty cycle limit was most often met between the retention times of 25–80 min which corresponded to the most feature-rich portion of the LCMS run. This indicated to us that the MS/MS spectra of many potential crosslinked peptides were not being acquired when using the conventional TopSpeed acquisition method (*i.e.* TopS, where the maximum number of the most intense peaks in an MS1 scan are acquired in a defined time period for each duty cycle), or the TopN acquisition method, where the N most-abundant peaks in an MS1 scan are acquired for each duty cycle.

**Fig. 3.**
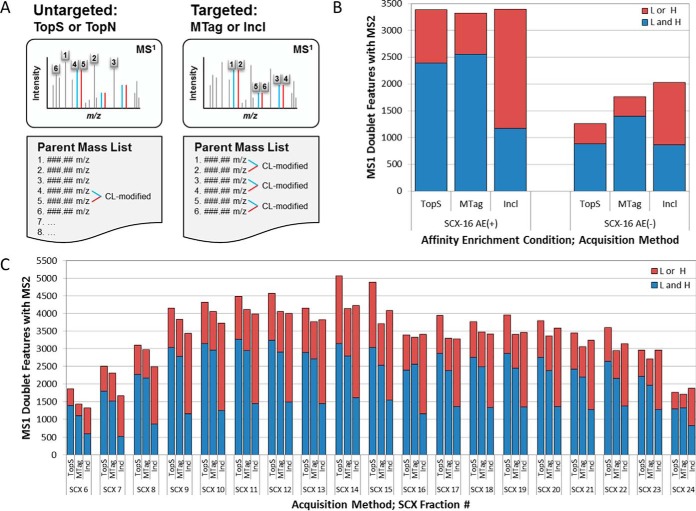
**Targeted acquisition improves the coverage of CL-modified peptides.** *A*, Diagram of untargeted and targeted acquisition methods. Both method types have precursors selected for MS/MS acquisition in order of MS1 signal intensity, but with a targeted method, the precursors must also be part of a Δ8.0502 Da doublet to be selected. *B*, A comparison of the number of Δ8.0502 Da doublet features found in the MS1 spectrum of soluble pre-fraction SCX fraction #16, which had corresponding MS/MS scans in the untargeted (TopS) and targeted (MTag, and Incl) acquisition methods, revealed that the MS/MS spectra of a larger number of crosslinker-modified precursors were acquired when targeted methods were used on sample fractions that had not been affinity enriched than on those fractions that had been affinity enriched. *C*, A comparison of the number of Δ8.0502-Da doublet features found in all SCX fractions that had been affinity enriched showed no benefit of targeted methods over the untargeted method for crosslinker-modified precursor acquisition. Here we show data from only the soluble pre-fraction.

Next, we compared the number of observed MS1 doublet features that were acquired as MS/MS spectra across three different data-dependent acquisition methods: the TopS method; the targeted mass difference (MTag) method; or inclusion lists derived from the MTag LCMS data from a prior injection (Incl) (Fig. 3*A*). In order to maximize the number of crosslinked peptides acquired in the MS/MS mode, a CL-specific acquisition strategy was developed and employed (supplemental Fig. S3). First, “MTag” LCMS data were collected for each SCX fraction. For this, an acquisition method was used that had a targeted mass difference filter set for the isotopic mass difference between the two forms of the crosslinker (delta) of the crosslinker—*e.g.* Δ8.0502 Da for CBDPS-H8/D8—along with a light-heavy partner intensity range set to 50–100. With this MTag method, the instrument is instructed to monitor MS1 scans for the presence of MS1 signals separated by the specified mass delta as they are being acquired, and to trigger MS2 acquisition of both the light and heavy partner precursor ions when the delta is observed (Fig. 3*B* and 3*C*). Because MS2 is only triggered when the mass delta is observed, the amount of duty cycle time the instrument expends acquiring MS2 scans for precursors that do not contain crosslinker is kept to a minimum (supplemental Fig. S2). This should result in the MS/MS acquisition of spectra from additional low-abundance crosslinker-modified precursor ions - ions which may have been otherwise not been acquired because of a low ranking in a standard TopN method parent mass list. In addition, we would expect to observe the acquisition of a larger number of unique MS1 peptide features when using an MTag acquisition method than when using a TopS method because the instrument can spend comparatively more time collecting MS1 scans with the MTag method.

To ensure that MS/MS spectra are acquired for as many crosslinker-modified precursors as possible from each sample/fraction in a single LCMS run, “Incl” LCMS data were also acquired for each fraction. Here a crosslinker-specific parent mass inclusion list was calculated using the MS1 data from the MTag acquisition. This was accomplished by processing the MS1 data from the MTag acquisition with a software pipeline that incorporates Hardklör (31), Krönik (32), and in-house scripts to generate .csv crosslinker-specific parent mass inclusion lists (supplemental Fig. S3). Calculation of these inclusion lists and construction of the inclusion list methods can be performed immediately after the MTag LCMS run is completed. With the Incl method, the instrument is instructed to monitor MS1 scans as they are being acquired for the presence of the specific masses in the parent mass list (which was calculated from the prior MTag run) and to trigger MS/MS acquisition of both the light and heavy partner precursor ions when observed.

Surprisingly, we found that the targeted methods showed no improvement in the number of MS1 doublet precursor ions acquired with MS/MS compared with untargeted methods for those samples in which enrichment was performed (Fig. 3*D*, 3*E*). In fact, the TopS method appeared to outperform both the MTag and Incl methods with respect to the number of MS1 doublet features acquired with both or only light or heavy isotopic precursor ion partners being acquired. The expected advantage of using a targeted acquisition method was *only* realized in the analysis of sample fractions that had not previously undergone the affinity enrichment step (Fig 3*B*, 3*C*). In this case, a 40% improvement in MS1 doublet acquisition was observed for the MTag method, and a 63% improvement was observed for the Incl method, compared with the TopS method. Presumably, the benefit realized by using targeted acquisition modes will increase together with the increasing complexity of the sample analyzed. This will be an important consideration when extending the technique to systems of increasing complexity (organelles, to cells, to tissues constituted of different cell types, etc.) or, potentially, in shortened analyses in which there is a lesser degree of sample pre-fractionation performed prior to enrichment in which we might expect to see greater performance improvements.

##### Integrating Crosslinker-specific Mass-spectral Feature Information for Improved Performance in Peptide-spectrum Match Validation

**Fig. 4.**
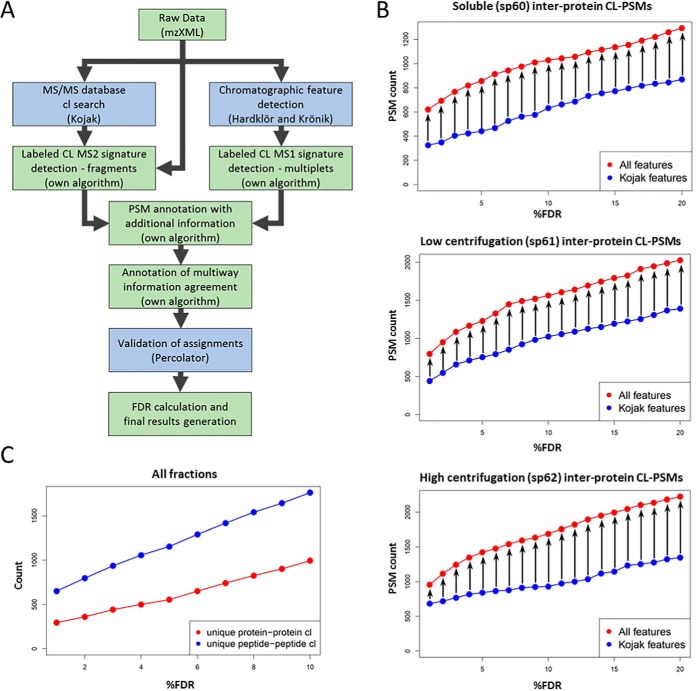
**Crosslinker-specific mass spectrum features improve crosslinker-modified peptide identification.** *A*, MS data is passed into our software pipeline that generates PSMs, extracts MS1 feature information, adds additional CL-specific feature information to each PSM, executes PSM validation, and returns validated PSMs. *B*, The number of identified inter-protein CL-PSMs as a function of %FDR are shown for PSM validation outputs from Percolator training with input using either the original set of Kojak PSM validation features, or the full set of PSM validation features from the data analysis pipeline. An increase in identified CL-PSMs was observed across all %FDR levels (0–20% shown). *C*, The total number of unique protein-protein interactions and unique residue-residue crosslink identifications in all datasets combined is shown as a function of %FDR.

The data-analysis pipeline integrates existing software tools and in-house-developed logic into a single tool (Fig. 4*A*). Briefly, MS data (.raw file format) were converted into mzXML format. Searches were performed using Kojak (34), which was configured to output all Kojak diagnostic files for each input mzXML file. For all of the searches and identifications, we used a protein database that we assembled based on the yeast mitochondrion proteome that had been previously investigated (28, 35, 40).

Concurrently the mzXML files were processed using Hardklör (31) and Krönik (32) software tools to produce a list of MS1 features. This list was then analyzed with our own algorithm to yield a list of crosslinking MS1 features, (*i.e.* paired MS1 features that exhibit the specific mass delta corresponding to the difference between heavy and light CBDPS crosslinker (*e.g.* 8.0502 Da)).

The search results from Kojak, as well as MS1 features from Hardklör/Kronik, were combined and further annotated with additional information on these features based on peptide-spectrum matches (PSMs) using our own algorithm. Specifically, we added to each PSM corresponding information from the Kojak diagnostic output including: preliminary and final scores and ranks for both the individual peptides, the Hardklör score for the precursor mass, the score difference between the best ranking and second best ranking PSM for all tested precursor masses, the label class (light or heavy) of the crosslink moiety, the relative mass error for the precursor as determined by Kojak and which exists in the mzXML, the total ion current, base peak m/z, and intensity for the MS2. We also annotated the PSMs with information derived from the list of paired MS1 features (*e.g.* the H/L intensity ratio for the isotopic partners, the retention time deltas for the isotopic partners, whether the isotopic pattern matches the expected pattern), in addition to information on the crosslinker-cleavage fragment ions in MS2 obtained directly from the mzXML file (*e.g.* the matched short and long crosslinker-cleavage fragment ions matched, the matched dead-end signature ions), and meta-PSM information (*e.g.* if a corroborating PSM exists for the corresponding isotopic partner).

A complete list of PSM features with descriptions is given in supplemental Table S4. In order to take advantage of the benefits that can be obtained by considering all of these feature dimensions simultaneously in a statistical validation of the PSMs, we used the popular semi-supervised machine learning algorithm Percolator (41). Each PSM is described by 41 feature dimensions and 5 Percolator-required dimensions (specid, Label, scannr, Peptide, Proteins). The new list of PSMs, now containing *additional* feature information, was then processed using Percolator for statistical validation.

A 20–30% increase was observed in confidently identified PSMs representing inter-protein crosslinks the additional feature information was included (Fig. 4*B*; supplemental material 2).

##### Overview of the Identifications with Respect to Fractionation

**Fig. 5.**
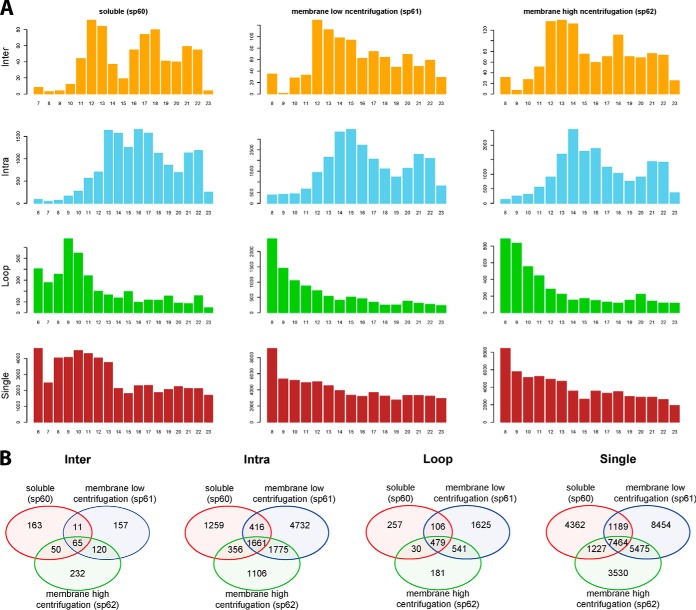
**Overview of the identifications.** *A*, The total number of (crosslinked-) peptide-spectrum-matches in each centrifugation fraction - three columns, and each SCX fraction -in numbers below each barplot. *B*, The overlap in identifications between the three centrifugation fractions. Crosslinks are divided into four types: inter- and intra-protein crosslinks, as well as loop and single peptide identifications.

The early SCX fraction contain predominantly single and loop peptides, whereas crosslinked peptides are appearing in the later SCX fractions (Fig. 5). The overlap in identification between the three centrifugation fractions shows the benefit of the pre-fractionation steps by centrifugation. Here we see a slight enrichment of inter-protein crosslinked peptides in the high centrifugation fraction, whereas intra-protein identifications are almost distributed between the high centrifugation and soluble fractions. Having a relatively higher number of identifications in the low centrifugation fraction, *i.e.* 2–3 times higher, in the intra-protein cross links as well as single and loop peptide identifications suggests that extra centrifugation steps could perhaps be beneficial for even better fractionation.

##### The Yeast Mitochondria Interactome

**Fig. 6.**
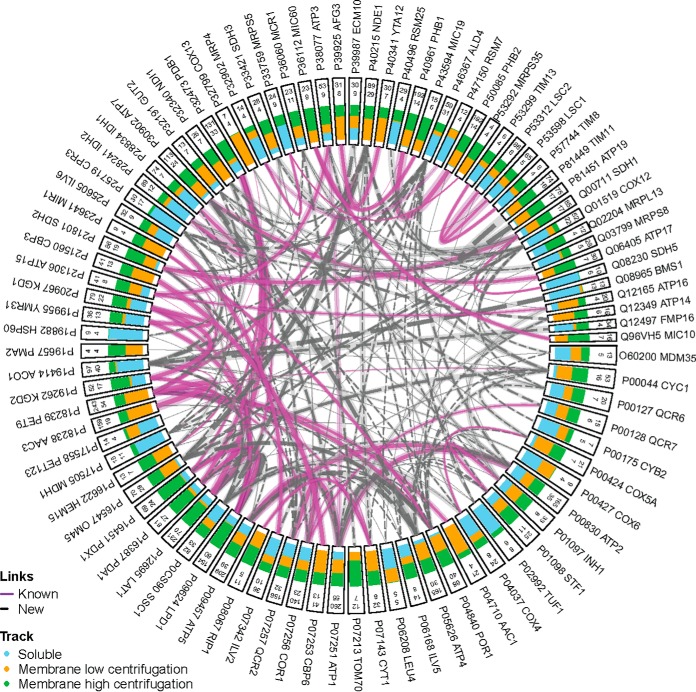
**Protein-protein interaction network analysis and sub-compartment localization of the identified crosslinks.** Unique inter-protein crosslinks identified at 2% FDR are represented for those proteins with a minimum of 4 unique residue-residue crosslinks. Classification of a protein interaction (edges) as “known” or “new” was based on the EMBL-EBI IntAct database of known yeast mitochondrial protein-interactions (retrieved: October 18, 2019) (45). Starting from the outside, each node is labeled with the UniProtKB accession number followed by the gene name. The number on the outside represents the total number of PSMs associated with that protein, followed by the number of unique residue-residue crosslinks associated with that protein. The green, orange, and blue bars inside each rectangle indicate sample pre-fractions in which the respective protein was identified. The width of the edges (*i.e.* the lines connecting nodes) represents the proportional number of all PSMs associated with the respective nodes with the number of validated PSMs between two proteins represented in semi-transparent highlighting and the number of unique residue-residue pairs represented by solid lines.

**Fig. 7.**
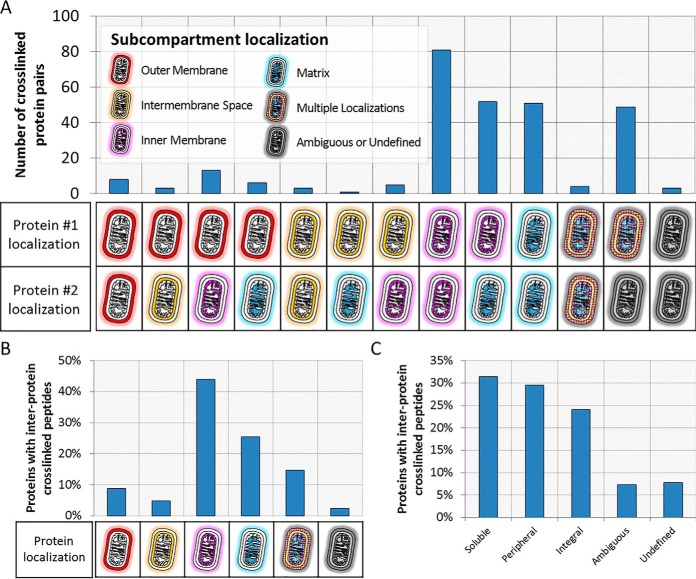
**Protein-protein interaction network analysis and sub-compartment localization of the identified crosslinks.** *A*, Distribution of sub-compartment localizations (based on Vögtle *et al.* (35)) for pairs of unique protein-protein interactions identified in this study (FDR 2%). *B*, Distribution of the protein classification for all proteins identified in inter-protein crosslinks. *C*, Distribution of sub-compartment localizations for proteins with identified inter-protein crosslinks (FDR 2%).

To demonstrate the analytical strategy described above to elucidate a protein-protein interactome, we analyzed highly purified yeast mitochondria where we found 751 non-redundant crosslinked inter-protein inter-peptide pairs that were identified (FDR 2%), involving 264 yeast mitochondrial proteins representing 338 unique protein-protein interactions (supplemental material 3, Table I, and Table II). These data provide structural insight into the protein-protein interactions of 20% of the proteins in the yeast mitochondrial proteome (Fig. 6) and, to our knowledge, represents the most comprehensive set of yeast mitochondrial protein-protein interactions determined in a single CLMS experiment to date. Furthermore, soluble, peripheral, and integral protein classes were approximately evenly represented in the interacting proteins accounting for 31%, 29%, and 24% of the proteins involved in PPIs, respectively (Fig. 7*B*; supplemental material 3) (35). Of the yeast mitochondrial interactions we identified, 71.7% were not previously described in the EMBL-EBI IntAct Molecular Interaction Database (downloaded on October 18, 2019) (Fig. 6*A*; supplemental material 3) (42, 43). In addition, we were also able to discover 185 previously unknown protein-protein interactions (Fig. 6, Table II, supplemental material 3). The distribution of the sub-compartment localizations of the proteins involved in the identified PPIs appears to make biological sense (Fig. 7*A*) (34). This data provides novel insights into the interactions of many mitochondrial proteins with soluble, peripheral and integral membrane proteins represented (Fig. 7*B*). Furthermore, 83% of the interacting proteins identified have previously described sub-compartment localizations and 17% previously ambiguous or undefined (Fig. 7*C*). The distribution of the sub-compartment localizations of the proteins involved in the identified PPIs appears to make biological sense (Fig. 7*A*) (35). The most observed subcompartment localization pairs were between inner-membrane proteins (81 PPIs), inner-membrane and matrix proteins (52 PPIs), and matrix proteins (51 PPIs). PPIs with protein localizations that would preclude interaction were observed infrequently or not at all (*e.g.* 6 outer-membrane to matrix PPIs were observed, no inter-membrane space to matrix were observed).

**Table I:** A comparison of recent mitochondria mass spectrometry-based crosslinking studies

Reference	This work	Schweppe et al. “Mitochondrial protein interactome elucidated by chemical crosslinking mass spectrometry.” Proceedings of the National Academy of Sciences 114.7 (2017): 1732–1737.	Liu et al. “The interactome of intact mitochondria by crosslinking mass spectrometry provides evidence for coexisting respiratory supercomplexes.” Molecular & Cellular Proteomics 17.2 (2018): 216–232.
Organism	Yeast	Mouse	Mouse
Material	Isolated mitochondria	Isolated mitochondria	Isolated mitochondria
Crosslinker	CBDPS	BDP-NHP	DSSO
Number of LC-MS runs	55 LC-MS runs	72 LC-MS runs	42 LC-MS runs for native condition crosslinking data
		11 biological replicates run in technical duplicate	21 SCX fractions for 2 biological replicates
Soluble-protein/membrane-protein fractionation	Yes	No	No
SCX fractionation	Yes	Yes	Yes
Affinity enrichment	Yes	Yes	No
Isotope labelling	Yes	No	No
Acquisition method types	TopN	ReACT (PMID:23413883)	TopN
Platform MS	Orbitrap Q Exactive HF	Velos-FTICR (custom-build)	Orbitrap Fusion
Non-redundant CL pairs	Inter:751	inter+intra: 2427	inter+intra: 3322
	Intra:9521		
Proteins Involved	Inter:251 (unambiguous)	inter+intra: 327	inter+intra: 359
	Intra:784 (unambiguous)		
	Total:811 (unambiguous)		
PPI's	Inter:338 (unambiguous)	459	Total: 885
	Intra:784 (unambiguous)		Intra: 276
			Inter:609
			(Not reported in manuscript, counted from supplementary materials)
FDR	2%	1.91%	2%

**Table II:** Identified protein-protein interactions with highest number of PSMs (a complete list is in the Supplemental Materials)

Protein A	Gene A	Protein B	Gene B	Total Number of PSMs	Previously reported in IntAct	Previously reported in SGD	Number of unique peptide-peptide IDs	Nr of PSMs in sp60 fraction (Soluble)	Nr of PSMs in sp61 fraction (Membrane low centrifugation)	Nr of PSMs in sp62 fraction (Membrane high centrifugation)
P40961	PHB1	P50085	PHB2	191	Yes	Yes	13	5	94	92
P18238	AAC3	P18239	PET9	157	Yes	No	18	0	117	40
P07256	COR1	P07257	QCR2	113	Yes	Yes	13	47	33	33
P07251	ATP1	P09457	ATP5	107	Yes	Yes	18	29	39	39
P12695	LAT1	P32473	PDB1	91	Yes	Yes	20	29	12	50
P81449	TIM11	P81451	ATP19	73	No	No	15	3	32	38
P00830	ATP2	P09457	ATP5	72	Yes	Yes	10	27	19	26
P28241	IDH2	P28834	IDH1	72	Yes	Yes	5	44	11	17
P09624	LPD1	P12695	LAT1	67	No	No	27	7	1	59
P05626	ATP4	P30902	ATP7	64	No	Yes	9	4	29	31
P53312	LSC2	P53598	LSC1	64	Yes	Yes	4	28	16	20
P21801	SDH2	Q00711	SDH1	54	Yes	Yes	7	7	20	27
P00830	ATP2	P07251	ATP1	51	Yes	Yes	9	17	16	18
P12695	LAT1	P16387	PDA1	46	Yes	Yes	13	9	4	33
P07253	CBP6	P21560	CBP3	41	Yes	Yes	13	0	27	14
P19262	KGD2	P20967	KGD1	40	Yes	Yes	9	19	3	18
P05626	ATP4	P07251	ATP1	38	No	Yes	6	2	22	14
P09624	LPD1	P16451	PDX1	35	Yes	Yes	10	7	2	26
P19414	ACO1	Q12497	FMP16	34	No	No	4	19	3	12
P16547	OM45	P40215	NDE1	33	No	No	11	0	21	12
P21306	ATP15	P38077	ATP3	31	Yes	Yes	4	10	8	13
P39925	AFG3	P40341	YTA12	30	Yes	Yes	7	0	21	9
P07342	ILV2	P25605	ILV6	28	Yes	Yes	7	12	8	8
P12695	LAT1	P16451	PDX1	27	No	No	10	2	0	25
P16387	PDA1	P32473	PDB1	27	Yes	Yes	8	5	4	18
P0CS90	SSC1	P39987	ECM10	26	No	No	7	7	10	9
P30902	ATP7	Q06405	ATP17	25	Yes	Yes	4	0	8	17
P40496	RSM25	Q03799	MRPS8	25	No	No	2	17	1	7
P05626	ATP4	P09457	ATP5	22	No	Yes	6	2	11	9
P04710	AAC1	P18239	PET9	21	Yes	Yes	4	0	18	3
P09624	LPD1	P19955	YMR31	20	Yes	Yes	8	11	1	8
P00830	ATP2	P05626	ATP4	19	No	Yes	3	7	9	3
P25349	YCP4	Q12335	PST2	19	Yes	Yes	3	2	16	1
P00044	CYC1	P16547	OM45	16	No	No	4	0	5	11
P19955	YMR31	P20967	KGD1	16	Yes	Yes	5	9	2	5
P05626	ATP4	Q12233	ATP20	15	No	Yes	1	2	7	6
P07143	CYT1	P40215	NDE1	15	No	No	1	0	10	5
P33421	SDH3	Q00711	SDH1	15	No	No	1	4	7	4
P53252	PIL1	Q12230	LSP1	15	Yes	Yes	3	0	9	6
P07251	ATP1	P0CS90	SSC1	14	No	No	3	6	4	4

##### Structural Validation of Crosslinks on Existing Structural Models

**Fig. 8.**
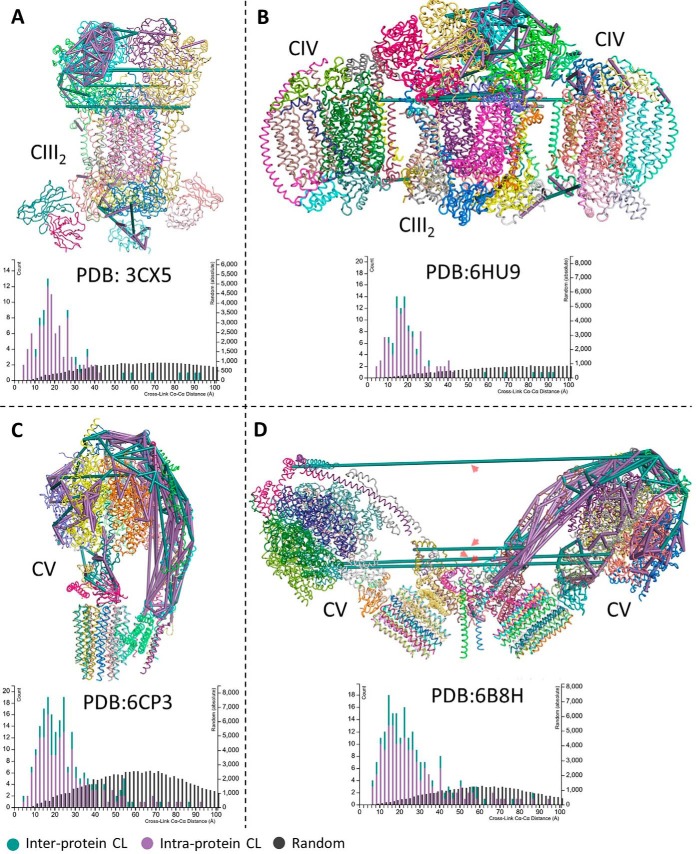
**Identified crosslinks mapped to PDB structures of yeast mitochondrial electron transport chain complexes and supercomplexes.** *A*, Mapping of identified crosslinks to complex III_2_ (PDB ID: 3CX5), *B*, complex V (PDB ID: 6CP3), *C*, respiratory super-complex III_2_IV_2_ (PDB ID: 6HU9), and *D*, to complex V dimer (PDB ID: 6B8H). All panels are accompanied with a histogram of observed Cα-Cα distance distributions *versus* distances of random possible links. Inter-protein crosslinks are shown as green lines and intra-protein crosslinks are shown as purple lines. In case a crosslink may be drawn multiple times (*e.g.* in each monomer of a homodimer) only the shortest constraint is shown. Red arrows in panel (*D*) label long crosslink distances ranging from 100 to 325 Å, however in alternative arrangements of two or more complex V dimers, the monomers are adjacent (side-by-side) with a distance of ∼130 Å between their rotational axis (44). In this arrangement an expected distances closer to 38 Å between the crosslinked sites may exist, however, PDB structures for this arrangement are not available.

We assessed the validity of our results by mapping the identified crosslinks to existing structural models of complexes involved in the mitochondrial electron transport chain available in the Protein Data Bank (PDB) database, *i.e.* 3CX5, 6HU9, 6CP3, and 6B8H. We also charted the observed Cα-Cα distance distributions *versus* distances of random possible links in each of these complexes. Fig. 8 shows the mapping of the identified inter- and intra-protein crosslinks to these four (super) complexes from the membrane high centrifugation fractions along with the histogram of the distances. The mapping shows good possible crosslinks with the majority being below the maximum Cα-Cα distance threshold of our crosslinker of 38 Å. Looking in details at these results, when mapping our identifications to the available PDB model of yeast mitochondrial ATP synthase (PDB: 6B8H), shown in Fig. 8*D*, where two ATP synthase monomers nagged in a V shape with an angle of 86°, we were initially worried about possible false identification with the long cross links between the two complexes ranging from 100 to 325 Å (labeled with red arrows in Fig. 8*D*). However, when four yeast mitochondrial ATP synthase dimers are adjacent side by side, the membrane becomes flatter resulting in parallel monomers organized side-by-side with 130 Å between their rotational axis (44). With an average diameter of 100 Å, one can calculate an expected distance of around 30 Å between the crosslinked residues (instead of 100–325 Å), which is very much in the range of our crosslinker. Unfortunately, there is no PDB structure with that formation of parallel monomers that we can map our crosslinks to supplemental Fig. S4 shows all identified crosslinks mapped to PDB structures of yeast mitochondrial electron transport chain complexes and supercomplexes for all sample pre-fractions.

## DISCUSSION

Chemical crosslinking combined with mass spectrometry is a valuable method for attaining structural information about proteins and identifying protein-protein interactions. Investigations on low complexity systems, for example purified proteins or protein complexes, are now becoming routine. However, applying this technique to complex systems, for example organelles or cells, still presents a variety of challenges. For example, these challenges involve technical aspects such as overcoming the inherently low abundance of crosslinked peptides which leads to limited detection in MS1 and concomitantly limited MS2 acquisition when using a typical shotgun proteomics workflow. These challenges also extend to various bioinformatic aspects, which include not only the efficient and confident identification of crosslinked peptides from MS2 spectra, but also exploiting all acquired data, including MS1 features, MS2 features, and meta-PSM features, in order to further improve identification of crosslinked peptides. To overcome these challenges, we have shown here that by utilizing an enrichable, isotopically labeled, MS-cleavable crosslinking reagent, targeted MS2 acquisition strategy, and a software pipeline designed to integrate CL-specific information we were able to improve the detection, acquisition, and identification of crosslinker-modified peptides and improve analysis of complex whole-proteome systems. This improved method was applied *in-organello* to isolated yeast mitochondria, and has allowed the detection of protein-protein interactions involving a sixth of the mitochondrial proteome. Moreover, 71.7% of these identified interactions comprise interactions not reported in the EMBL-EBI IntAct Molecular Interaction Database (43, 45), whereas when comparing with *Saccharomyces* Genome Database (SGD) database (46)—which is better annotated—61% identified interactions were not reported. However, it is important to mention that the annotation of interactions in all databases are not complete and lag behind the literature, so for example interactions related to Pyruvate Dehydrogenase (PDH) or Succinate Dehydrogenase (SDH) complexes are well known and identified in our data, but do not appear neither in IntAct nor SGD. A validation of the identified crosslinks by mapping to existing structural models of complexes involved in the mitochondrial electron transport chain available from PDB showed good agreement. In all four (super) complexes used, the Cα-Cα distance distributions agreed to with the expectation of the used chemical crosslinker, *i.e.* distances of 38 Å and less. There is no PDB model available for yeast mitochondrial ATP synthase with four or more monomers. Mapping to the available one dimer (PDB: 6B8H) produces misleading results suggesting inter-monomer crosslinks of 100 to 325 Å in length. However, it has been shown previously (44, 47) that when four or more dimers are adjacent side by side, the membrane flattens resulting in monomers organized in parallel side by side with 130 Å between their rotational axis. In this arrangement and with a calculated distance of around 30 Å, it is very likely that the identified inter-complex crosslinks (red arrows Fig. 8*D*) are correct. The presented *ex vivo* crosslinking analytical approach is suitable for proteome-wide applications and provides a technical foundation that will yield insights into condition-specific protein conformations, protein-protein interactions, system-wide protein function or dysfunction, and diseases. The software modules developed in-house are available from http://bioinformatics.proteincentre.com/Qualis-CL/ and from the authors.

### Conflicts of Interest

The authors have declared a conflict of interest. EVP and CHB are co-founders of Creative Molecules Inc. The other authors declare no competing interests.

## DATA AVAILABILITY

The mass spectrometry proteomics data have been deposited in the ProteomeXchange Consortium via the PRIDE (48) partner repository with the dataset identifier PXD014055 and PXD017066. Peptide assignments can be viewed using Kojak Viewer. Instructions are available in supplemental material 4.

## Supplementary Material

Supplemental Materials

Supplemental Figures and Tables

Supplemental Material 1

Supplemental Material 2

Supplemental Material 3

Supplemental Material 4

## Footnotes

^1^ The abbreviations used are:

CLMS: chemical crosslinking and mass spectrometry
MS: mass spectrometry
CBDPS: cyanurbiotindipropionylsuccinimide.
