# Supplementary material for: Improving Identification of In-organello Protein-Protein Interactions Using an Affinity-enrichable, Isotopically Coded, and Mass Spectrometry-cleavable Chemical Crosslinker
Source: Mol Cell Proteomics. 2020 Feb 12;19(4):624–39. doi: 10.1074/mcp.RA119.001839 (PMC7124466; doi:10.1074/mcp.RA119.001839)
Supplement: Supplemental Materials [file RA119.001839_index.html]

Supplement to Improving identification of in-organello protein-protein interactions using an affinity-enrichable, isotopically-coded, and mass spectrometry-cleavable chemical crosslinker | Molecular & Cellular Proteomics

## Supplemental Data

- Supplemental Figures and Tables - Supplemental Material Figures S1-S4. Supplemental Material Table S1. Hardkl&#x00F6;r parameters. Supplemental Material Table S2. Kr&#x00F6;nik parameters. Supplemental Material Table S3. Kojak parameters. Supplemental Material Table S4. Description of all features used to represent PSMs. Supplemental Material Table S5. Percolator parameters.
- Supplemental Material 1 - FASTA sequence database used for Kojak searches.
- Supplemental Material 2 - List of (crosslinked) peptide-spectrum matches identified
- Supplemental Material 3 - List of protein-protein interactions identified. Excel table (.xlsx) of protein-protein interactions identified in QExactiveHF SP60, 61, 62 SCX datasets at 2% FDR with comparison to known protein sub-compartment localizations.
- Supplemental Material 4 - Instructions for viewing annotated spectra using Kojak Spectrum Viewer
