# Supplementary material for: Improving Identification of In-organello Protein-Protein Interactions Using an Affinity-enrichable, Isotopically Coded, and Mass Spectrometry-cleavable Chemical Crosslinker
Source: Mol Cell Proteomics. 2020 Feb 12;19(4):624–39. doi: 10.1074/mcp.RA119.001839 (PMC7124466; doi:10.1074/mcp.RA119.001839)
Supplement: Supplemental Figures and Tables [file 156666_1_supp_459899_q48453.docx]

# Supplemental Information – Figures and Tables

# **Improving identification of *in-organello* protein-protein interactions using an affinity-enrichable, isotopically-coded, and mass spectrometry-cleavable chemical crosslinker**

Karl A. T. Makepeace^1,2†^, Yassene Mohammed^2,3†^, Elena L. Rudashevskaya^4†^, Evgeniy V. Petrotchenko^2,5^, F.-Nora Vögtle^6,7^, Chris Meisinger^6,7^, Albert Sickmann^4*^, and Christoph H. Borchers^1,2,5,8*^

1. Department of Biochemistry and Microbiology, University of Victoria, Petch Building, Room 270d, 3800 Finnerty Rd., Victoria, BC V8P 5C2, Canada

2. University of Victoria - Genome British Columbia Proteomics Centre, #3101-4464 Markham Street, Vancouver Island Technology Park, Victoria, BC V8Z7X8, Canada

3. Center for Proteomics and Metabolomics, Leiden University Medical Center, Albinusdreef 2, 2333 ZA, Leiden, The Netherlands

4. Leibniz Institut für Analytische Wissenschaften - ISAS - e.V., Dortmund, Germany

5. Segal Cancer Proteomics Centre, Lady Davis Institute, Jewish General Hospital, McGill University, Montreal, Quebec, H3T 1E2, Canada

### 6. Institute of Biochemistry and Molecular Biology, ZBMZ, Faculty of Medicine, University of Freiburg, Freiburg, Germany

7. Signalling Research Centres BIOSS and CIBSS, University of Freiburg, Germany

8. Gerald Bronfman Department of Oncology, Jewish General Hospital, Montreal, Quebec, H3T 1E2, Canada

^†^these authors contributed equally to the manuscript

*Corresponding authors:

Christoph H. Borchers (christoph@proteincentre.com)

Albert Sickmann (sickmann@isas.de)

## Supplemental Figures

## Supplemental Tables

**Supplemental Information Table S1.** Hardklör parameters.

**Supplemental Information Table S2.** Krönik parameters.

**Supplemental Information Table S3.** Kojak parameters.

**Supplemental Information Table S4.** Description of all features used to represent PSMs.

**Supplemental Information Table S5.** Percolator parameters.


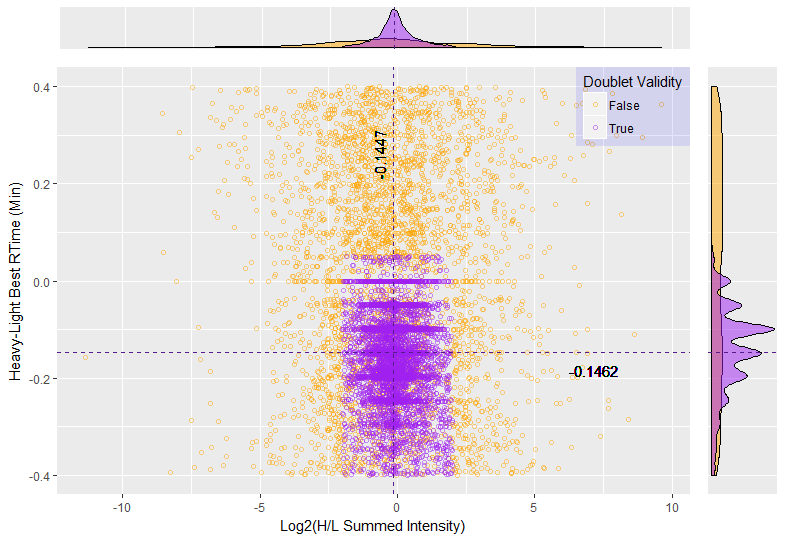


**Supplemental Information Figure S1.** MS1 Δ8.0502 Da doublet features identified in Kronik output. Criteria for classifying an MS1 feature from Krönik output as a true doublet was that the mass difference between the light and heavy monoisotopic peaks for the pair of MS1 features are 8.050213952 Da +/-0.01 Da, that the heavy-isotopic peak has a maximum intensity that occurs at a retention time that is between -0.4 min and 0.05 min of the maximum intensity observed for the light-isotopic peak, and that the maximum intensities observed for both light and heavy isotopic peaks are each greater than or equal to 25000 intensity units. The doublet identification and true/false classification is illustrated here using data from Lumos_02558.raw. The median log_2_(H/L) ratio for the summed isotopic-partner intensities for those MS1 doublet features classified as “true” was 0+/-2. A multimodal frequency distribution in the RTime H-L is observed for these features and is hypothesized to be related to the to the TopN spacing.

**Supplemental Information Figure S2.** The percent of total duty cycles between retention time 20 min and 80 min with N MS2 scans (TopN spacing) for soluble pre-fraction SCX fraction #16 T is represented for each acquisition method (TopS, MTag, or Incl). The duty cycle is frequently reaching the cycle time limits with TopS method (here limited by TopN spacing=10 or time=3 sec) during what we expect to be the portion of the LC-MS analysis that is most abundant in CL-modified precursors (retention times between 20 and 80 minutes). With the use of a CL-specific targeted acquisition methods (i.e. MTag or Incl), the duty cycle reaches the cycle limit more infrequently than with the untargeted acquisition method (i.e. TopS).

**
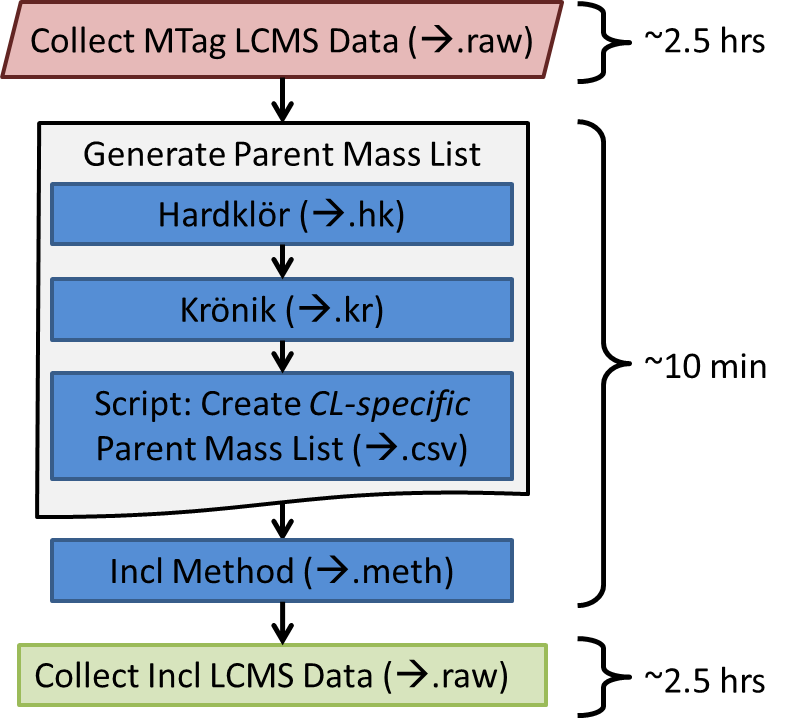
**

**Supplemental Information Figure S3.** Schematic of method and approximate time required for acquisition of MTag and Incl datasets for a sample fraction.


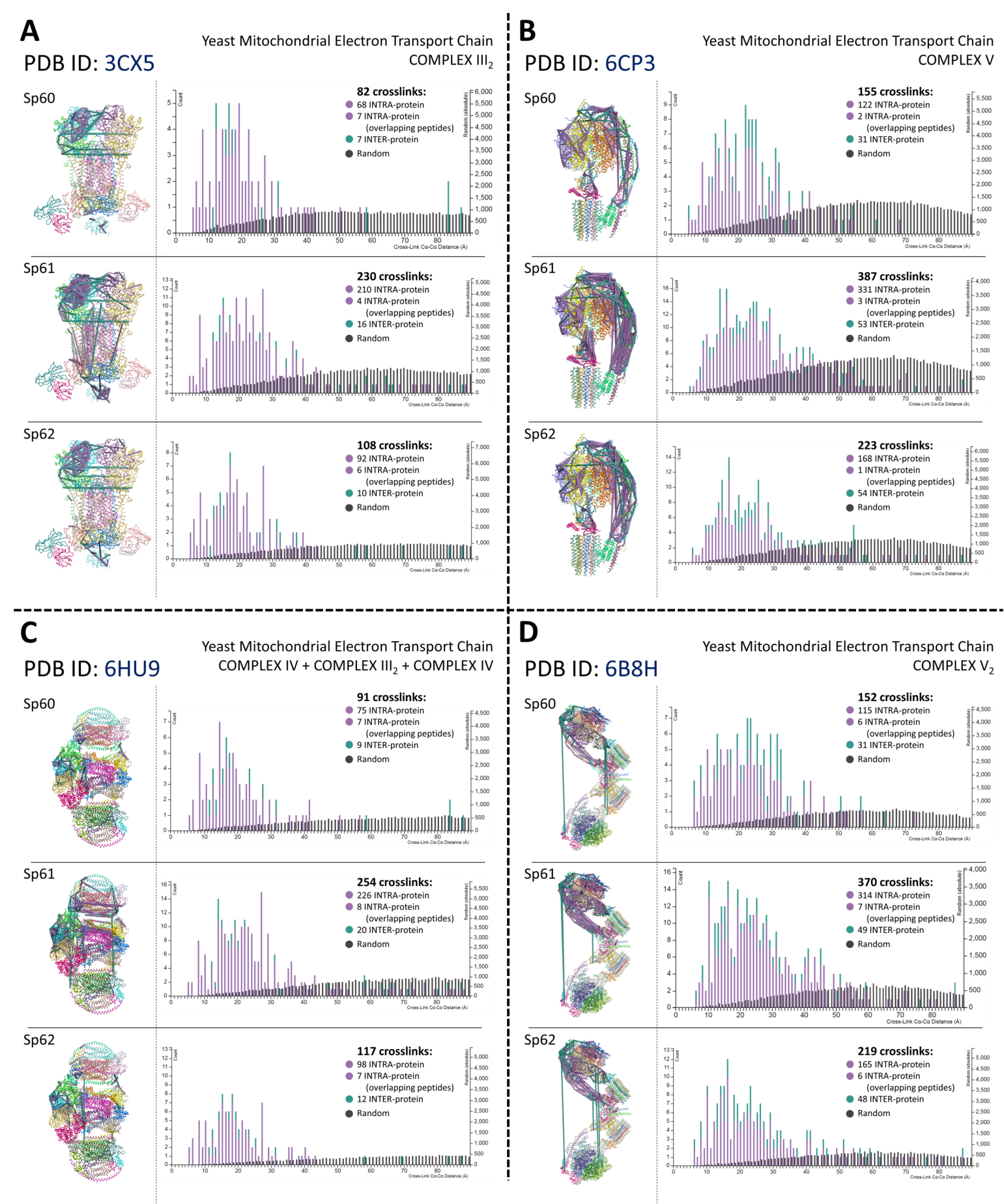


**Supplemental Information Figure S4.** Identified crosslinks mapped to PDB structures of yeast mitochondrial electron transport chain complexes and supercomplexes for all sample pre-fractions. (A) Mapping of identified crosslinks to complex III_2_ (PDB ID: 3CX5), (B) complex V (PDB ID: 6CP3), (C) respiratory super-complex III_2_IV_2_ (PDB ID: 6HU9), and (D) to complex V dimer (PDB ID: 6B8H). Panels are accompanied with a histogram of observed Cα-Cα distance distributions versus distances of random possible links. Inter-protein crosslinks are shown as green lines and intra-protein crosslinks are shown as purple lines. In cases in which a crosslink may be drawn multiple times (e.g. in each monomer of a homodimer) only the shortest constraint is shown.

# Supplemental Information Tables

**Supplemental Information Table S1.** Hardklör parameters.

| Parameter | Setting |
| --- | --- |
| instrument | Orbitrap |
| resolution | 120000 |
| centroided | 0 |
| ms_level | 1 |
| scan_range_min | 0 |
| scan_range_max | 0 |
| signal_to_noise | 2 |
| sn_window | 250 |
| static_sn | 0 |
| boxcar_averaging | 0 |
| boxcar_filter | 0 |
| boxcar_filter_ppm | 5 |
| mz_min | 0 |
| mz_max | 0 |
| smooth | 0 |
| algorithm | Version2 |
| charge_algorithm | Quick |
| charge_min | 1 |
| charge_max | 9 |
| correlation | 0.95 |
| averagine_mod | 0 |
| mz_window | 5.25 |
| sensitivity | 2 |
| depth | 2 |
| max_features | 12 |
| distribution_area | 1 |
| xml | 0 |

**Supplemental Information Table S2.** Krönik parameters.

| Parameter | Setting |
| --- | --- |
| -c | 5.0 |
| -d | 3 |
| -g | 1 |
| -m | 10000.0 |
| -n | 400.0 |
| -p | 10.0 |

**Supplemental Information Table S3.** Kojak parameters.

| Parameter | Setting |
| --- | --- |
| threads | 7 |
| database | ...\UP-Mitos-20161223-TDrev-K.fasta |
| export_percolator | 1 |
| export_pepXML | 1 |
| percolator_version | 2.08 |
| enrichment | 0 |
| instrument | 0 |
| MS1_centroid | 0 |
| MS2_centroid | 0 or 1 depending on dataset |
| MS1_resolution | 120000 |
| MS2_resolution | 60000 |
| cross_link | nK; nK; 509.097364; CBDPS_Light |
| cross_link | nK; nK; 517.147578; CBDPS_Heavy |
| mono_link | nK; 526.1238898 |
| mono_link | nK; 527.107929 |
| mono_link | nK; 534.1741038 |
| mono_link | nK; 535.158143 |
| modification | M; 15.9949 |
| modification_protC | 0 |
| modification_protN | 0 |
| diff_mods_on_xl | 1 |
| max_mods_per_peptide | 1 |
| mono_links_on_xl | 1 |
| enzyme | [KR]\|{P} |
| fragment_bin_offset | 0 |
| fragment_bin_size | 0.01 |
| ion_series_A | 0 |
| ion_series_B | 1 |
| ion_series_C | 0 |
| ion_series_X | 0 |
| ion_series_Y | 1 |
| ion_series_Z | 0 |
| decoy_filter | decoy |
| isotope_error | 1 |
| max_miscleavages | 3 |
| max_peptide_mass | 8000 |
| min_peptide_mass | 600 |
| max_spectrum_peaks | 0 |
| ppm_tolerance_pre | 3 |
| prefer_precursor_pred | 2 |
| spectrum_processing | 1 |
| top_count | 300 |
| truncate_prot_names | 0 |
| turbo_button | 0 |

**Supplemental Information Table S4.** Description of all features used to represent PSMs.

| **#** | **Feature Name** | **Category** | **Description** | **Datatype** |
| --- | --- | --- | --- | --- |
| 1 | specid | pIN | Unique PSM identifier | string |
| 2 | Label | pIN | Target/Decoy PSM label (1 = Target PSM; -1 = Decoy PSM) | integer |
| 3 | scannr | pIN | MS2 scan number for PSM | integer |
| 4 | Score | Score | The Kojak cross-correlation score | decimal |
| 5 | dScore | Score | The difference between the reported PSM score and the next best PSM score as reported in the original Kojak output | decimal |
| 6 | NormRank | Score | The sum of the Peptide #1 and Peptide #2 ranks in the first scoring pass of the Kojak algorithm | integer |
| 7 | PPScoreDiff | Score | The score contribution of only the lower scoring peptide (always reported as "pep_b") in a cross-link | decimal |
| 8 | Charge | Precursor | Precursor ion charge state (determined by Kojak) | integer |
| 9 | Mass | Precursor | Theoretical neutral mass of the PSM (value returned from Kojak) | decimal |
| 10 | PPM | Precursor | Difference (in parts per million) between the PSM mass (Kojak) and the Obs Mass (Kojak) | decimal |
| 11 | LenShort | Sequence | The residue count of the shortest of Peptide #1 and Peptide #2. | integer |
| 12 | LenLong | Sequence | The residue count of the longest of Peptide #1 and Peptide #2. | integer |
| 13 | LenSum | Sequence | The summed residue count of Peptide #1 and Peptide #2. | integer |
| 14 | pep_a_rank_1st_pass1 | Diagnostic Info | The rank of Peptide #1 based on the first reported score in the Kojak diagnostic output | decimal |
| 15 | pep_a_pass1_score | Diagnostic Info | The first reported score for Peptide #1 in the Kojak diagnostic output | decimal |
| 16 | pep_a_pass2_score | Diagnostic Info | The second reported score for Peptide #1 in the Kojak diagnostic output | decimal |
| 17 | pep_b_rank_1st_pass1 | Diagnostic Info | The second reported score for Peptide #1 in the Kojak diagnostic output | decimal |
| 18 | pep_b_pass1_score | Diagnostic Info | The second reported score for Peptide #1 in the Kojak diagnostic output | decimal |
| 19 | pep_b_pass2_score | Diagnostic Info | The second reported score for Peptide #1 in the Kojak diagnostic output | decimal |
| 20 | hardklor_prec_score | Diagnostic Info | The second reported score for Peptide #1 in the Kojak diagnostic output | decimal |
| 21 | dscore | Diagnostic Info | The difference between the reported PSM score and the next best PSM score as calculated from the best scoring PSM score minus the next best scoring PSM score in the Kojak diagnostic output. | decimal |
| 22 | l_or_h | Isotopic-coding | Label for the isotopically light (="-1") or isotopically heavy (="1") crosslinker moiety | integer |
| 23 | rel_err_mass_meas_koj | Precursor | The mass difference between the theoretical PSM Mass and the precursor mass as reported by Kojak | decimal |
| 24 | rel_err_mass_mzxml_calc | Precursor | The mass difference between the theoretical PSM Mass and the precursor mass as reported in the mzXML | decimal |
| 25 | ms2_feat_pep_a | CL-cleavage | The sum of booleans for matched crosslinker-cleavage ions for Peptides #1 for charge states of precursor z-1 (possible values = 0,1,2) | integer |
| 26 | ms2_feat_pep_b | CL-cleavage | The sum of booleans for matched crosslinker-cleavage ions for Peptides #2 for charge states of precursor z-1 (possible values = 0,1,2) | integer |
| 27 | log_ms2_totIonCurrent | Precursor | Log_10_ of the total ion current in the MS2 scan | decimal |
| 28 | ms2_basePeakMZ | Precursor | The base peak intensity in the MS2 scan | decimal |
| 29 | log_ms2_basePeakIntensity | Precursor | Log_10_ of the base peak intensity in the MS2 scan | decimal |
| 30 | de_sig_expectation_match | CL-cleavage | Whether the observed matching of the MS2 DE-signatures match the expectation for the PSM. | boolean |
| 31 | log2_int_most_l_int_most_h | Isotopic-coding | The binary logarithm of the heavy/light ratio of the maximum XIC intensity observed for the precursor MS1 feature (+/- 1 min). If PSM DX Level (i.e. the number of isotopically-coded moieties that exist for the PSM) > 1 then the MS1 signals corresponding to entirely light and entirely heavy isotopic forms are used in this calculation. If PSM DX Level = 0 then 0 is returned. | decimal |
| 32 | rt_diff_most_l_most_h | Isotopic-coding | The retention time (sec) difference from the observed light isotopic partner XIC maximum intensity to the heavy isotopic partner XIC maximum intensity. If PSM DX Level > 1 then the MS1 signals corresponding to entirely light and entirely heavy isotopic forms are used in this calculation. If PSM DX Level = 0 then 0 is returned. | decimal |
| 33 | prec_charge_mzxml_best_kj_agree | Precursor | Whether the best scoring precursor charge determination in the Kojak diagnostic output match the precursor charge reported in the mzXML. | boolean |
| 34 | prec_charge_kj_best_kj_agree | Precursor | Whether the best scoring precursor charge determination in the Kojak diagnostic output match the best scoring PSM in the Kojak diagnostic output. | boolean |
| 35 | heavy_mods_pos_in_multiplits_agree | Isotopic-coding | Whether the acquired precursor match the appropriate isotopic MS1 precursor signal for the PSM. | boolean |
| 36 | koj_kr_lh_agree | Isotopic-coding | Whether the isotopic label assignment for heavy and light by Kojak PSM and Kronik agree. | boolean |
| 37 | iso_part_psm | Isotopic-coding | The number of unique isotopic partners that match the PSM. If no isotopic partners are possible then equals -1. | boolean |
| 38 | charge2 | Precursor | Precursor ion charge state = 2 (determined by Kojak) | boolean |
| 39 | charge3 | Precursor | Precursor ion charge state = 3 (determined by Kojak) | boolean |
| 40 | charge4 | Precursor | Precursor ion charge state = 4 (determined by Kojak) | boolean |
| 41 | charge5 | Precursor | Precursor ion charge state = 5 (determined by Kojak) | boolean |
| 42 | charge6 | Precursor | Precursor ion charge state = 6 (determined by Kojak) | boolean |
| 43 | charge7 | Precursor | Precursor ion charge state = 7 (determined by Kojak) | boolean |
| 44 | charge8 | Precursor | Precursor ion charge state = 8 (determined by Kojak) | boolean |
| 45 | Peptide | pIN | Peptide #1 and Peptide #2 strings combined (for pIN) | string |
| 46 | Proteins | pIN | Non-redundant tab-separated list of all proteins listed in Protein #1 and Protein #2 (for pIN) | string |

**Supporting information Table S5.** Percolator parameters.

| Parameter | Setting |
| --- | --- |
| -w | …\[filename].SVMweights.txt |
| -U | n/a |
| -Y | n/a |
| -F | 0.001 |
