# Supplementary material for: Improving Identification of In-organello Protein-Protein Interactions Using an Affinity-enrichable, Isotopically Coded, and Mass Spectrometry-cleavable Chemical Crosslinker
Source: Mol Cell Proteomics. 2020 Feb 12;19(4):624–39. doi: 10.1074/mcp.RA119.001839 (PMC7124466; doi:10.1074/mcp.RA119.001839)

**Instructions for viewing annotated spectra using the Kojak Spectrum Viewer**

1. Download the Kojak Spectrum Viewer application (available at: <http://www.kojak-ms.org/viewer/index.html>)

2. Download “.mzxml” and corresponding “.pep.xml” files from the PRIDE data repository (project web address: https://www.ebi.ac.uk/pride/archive/projects/PXD017066/).

- e.g. QExactiveHF02_05787_SP0061_SCX16-A_TopN.mzxml and QExactiveHF02_05787_SP0061_SCX16-A_TopN.pep.xml

3. Keep both “.mzxml” and “.pep.xml” files in the same file directory. E.g.:


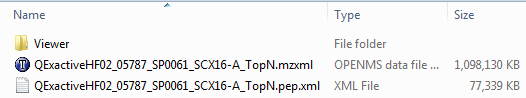


4. Launch the Kojak Spectrum Viewer application (KojakSpectrumViewer.exe) and use it to open the “.pep.xml” file. E.g.:


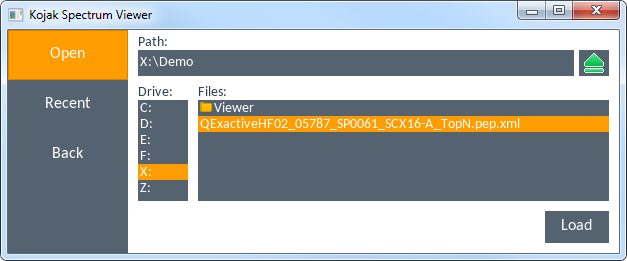


5. Turn on annotation of charge state 2+ and 3+ fragment ions by toggling on the corresponding boxes at the top of the right-side window pane. Navigate to the spectrum you would like to evaluate.


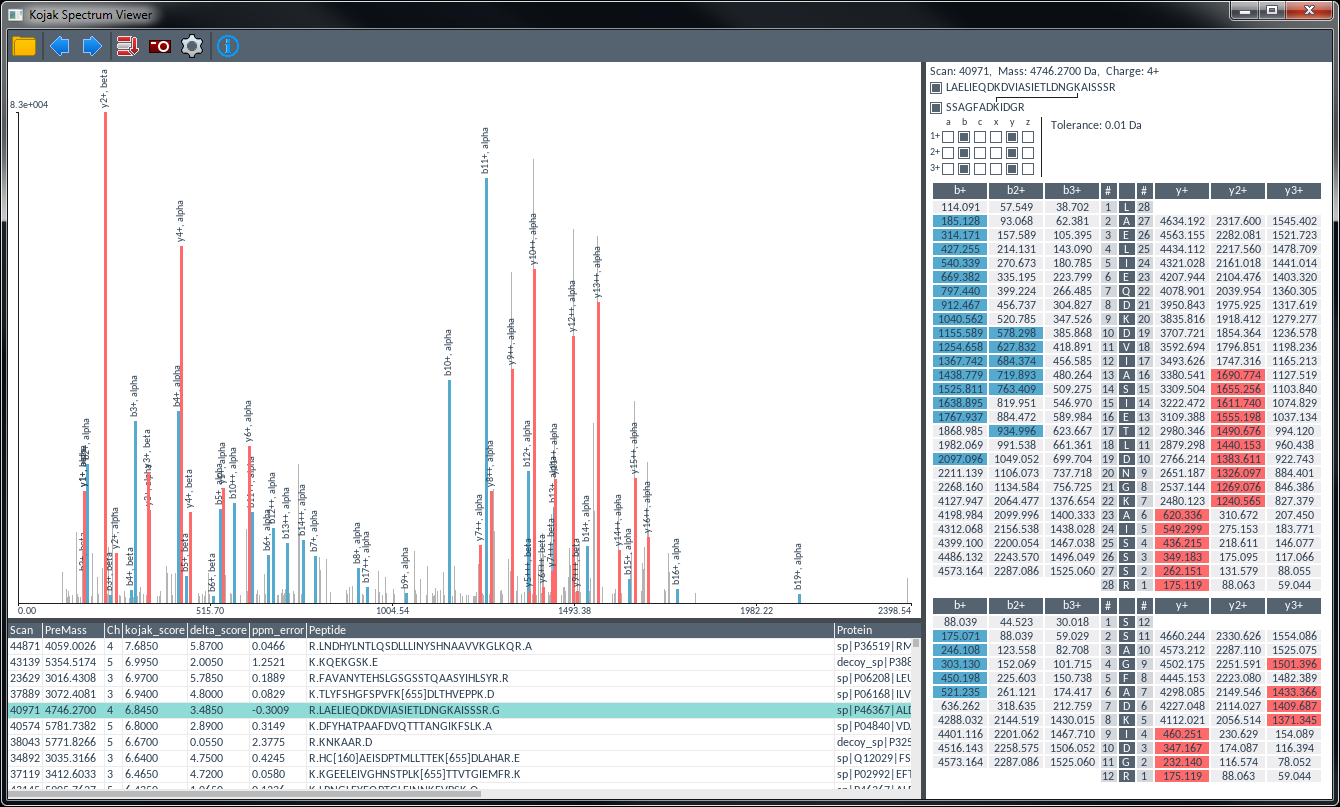

Supplement: Supplemental Material 4 [file 156666_1_supp_459948_q485m9.docx]
